# Fasting Plasma Glucose and Coronary Heart Disease in a Rural Population of North Henan, China

**DOI:** 10.1155/2020/2348583

**Published:** 2020-10-10

**Authors:** Quan Guo, Fei Lin, Yi Liu, Yang Li, Xue-Hui Wang, Zhi-Gang Chen, Feng-Hua Lv, Yong-Chun Zhang, Yu Yuan, Guo-An Zhao

**Affiliations:** Department of Cardiology, First Affiliated Hospital of Xinxiang Medical University, Xinxiang 453100, Henan, China

## Abstract

Even in individuals without diabetes, the incidence of coronary heart disease (CHD) increases with the rise in fasting plasma glucose (FPG); however, the threshold of FPG for CHD in rural areas of China is unclear. We retrospectively examined 2,987 people. Coronary angiography records were used to determine the presence of CHD as well as its severity. Risk factors for CHD and the relationship between different levels of FPG and CHD were analyzed. After adjusting for age, hypertension, dyslipidemia, smoking, drinking, chronic kidney disease, and previous ischemic stroke, the incidence of CHD in nondiabetic women began to increase when FPG exceeded 5.2 mmol/L (odds ratio (OR) = 1.438, 95% confidence interval (CI) = 1.099–1.880, *p*=0.008), and the degree of coronary artery lesions also became more severe (OR = 1.406, 95% CI = 1.107–1.788, *p*=0.005). However, no such correlations were found in nondiabetic men. In conclusion, among the nondiabetic women in rural areas of northern Henan, both the incidence of CHD and the severity of lesions increased when FPG levels were greater than 5.2 mmol/L, while no significant correlation between FPG and CHD was observed in diabetes-free men.

## 1. Introduction

Coronary heart disease (CHD) is a prevalent systemic disease worldwide. It places a heavy economic burden on countries and seriously affects the quality of life. Type 2 diabetes has long been recognized as an independent risk factor for CHD as excessive blood glucose levels can impair endothelial function [1]. However, the pathology of diabetes is a long-term chronic process. Prior to the diagnosis of diabetes, patients would have already developed insulin resistance and blood glucose metabolism disorders, a condition known as prediabetes, which involves impaired fasting glucose (IFG) levels and impaired glucose tolerance [2, 3]. The American Diabetes Association (ADA) defines IFG as a fasting plasma glucose (FPG) level of 5.6–6.9 mmol/L, while the World Health Organization (WHO) defines it as an FPG level of 6.1–6.9 mmol/L [4]. Most research studies on prediabetes and cardiovascular disease have been based on these two definitions, but the results have not been uniform [4–7]. The Framingham cohort study demonstrated that there was a positive correlation between the incidence of IFG and cardiovascular events in women, regardless of the definition, but this correlation did not extend to men [8]. A study in Asian populations found an increase in the incidence of cardiovascular events when FPG exceeded 4.9 mmol/L but did not find any differences between race and sex [9]. Indeed, different regions and races should have their own sets of blood glucose values for defining IFG. A meta-analysis found no increase in CHD risk in subjects without diabetes at baseline when FPG concentrations ranged between 3.9 mmol/L and 5.6 mmol/L. However, none of the 102 studies published were from China, and the selected populations were from urban areas [10]. Relative to urban populations, rural populations are typically less educated, have poorer health compliance, lack regular medical examinations, have lower sanitation standards, and experience different climates [11]. These differences may account for the discrepancies in their cardiovascular risk factor profiles and the incidence of cardiovascular events [12]. For example, in this research, women in rural areas of northern Henan were found to have lower smoking rates than those of the women in the Framingham cohort study. In recent years, the mortality rate of CHD in rural China has exceeded that of the urban population [13]. However, few studies have targeted such population groups. Moreover, the relationship between FPG and coronary atherosclerotic heart diseases in rural areas of northern Henan has not been reported. Therefore, the purpose of this investigation was to retrospectively examine patients in rural areas of northern Henan. We sought to explore the impact of ADA- and WHO-defined IFG on the incidence and severity of CHD and determine whether a more suitable IFG threshold exists for the rural populations of Henan.

## 2. Materials and Methods

### 2.1. Study Sample

All procedures were approved by the Ethics Committee of the First Affiliated Hospital of Xinxiang Medical College (Approval number 2018118). All data were obtained from the Henan Clinical Data and Sample Research Center for Cardiovascular Diseases database, which was established by the hospital and contains the medical records of all inpatients of the cardiovascular department since January 2016. A total of 3,884 patients from rural areas who underwent coronary angiography between January 2016 and July 2018 were selected from the database. Subsequently, we did not include patients without the necessary indicators for this analysis (417 cases), with previous stent implantation (272 cases), with coronary artery bypass grafting (1 case), with hyperthyroidism (58 cases) or hypothyroidism (14 cases), with severe liver dysfunction (36 cases), with FPG levels less than 3.9 mmol/L (66 cases), who are pregnant (0 cases), who consumed oral steroidal anti-inflammatory drugs (0 cases), and who had malignant tumors (32 cases) or a serious myocardial bridge (1 case) in the final analysis. A total of 2,987 patients were eventually included, and all participants had complete information, including name, hospital number, personal code, age, sex, FPG, liver function, kidney function, blood pressure, smoking history, drinking history, past medical history, blood lipids (including total cholesterol, triglycerides, high-density lipoprotein cholesterol, and low-density lipoprotein cholesterol), coronary angiography records, and Gensini scores (GS). There were no demographic differences between the excluded data and the final analyzed data.

### 2.2. Baseline and Definition

All patients underwent coronary angiography at the Heart Center of the First Affiliated Hospital of Xinxiang Medical College between January 2016 and July 2018. Two or more experienced physicians assessed the angiographic results. CHD was defined as stenosis of ≥ 50% in at least one major or branch vessel. The degree of coronary artery lesions was divided into 4 groups according to the GS [14]: normal (GS < 1), mild lesions (1 ≤ GS < 20), moderate lesions (20 ≤ GS < 49), and severe lesions (GS ≥ 49).

All patients underwent a venous blood draw for glucose measurement after fasting for more than 8 hours. Those with FPG ≥7.0 mmol/L, a history of diabetes, or currently using hypoglycemic drugs (including oral hypoglycemic agents and insulin) were classified as having diabetes. IFG was divided into IFG-ADA (5.6–6.9 mmol/L), IFG-WHO (6.1–6.9 mmol/L) [4], or other specific cut-off points, according to the different definitions.

Hypertension was defined as a history of hypertension, the current use of oral antihypertensive drugs, or a systolic blood pressure ≥ 140 mmHg or diastolic blood pressure ≥ 90 mmHg at admission.

Smoking was defined as current or previous smoking habits involving an average of more than one cigarette per day for more than 6 months.

Drinking was defined as a current or past history of alcohol consumption, with an average daily amount ≥ 40 g for a period longer than 6 months.

Dyslipidemia was considered in patients with triglycerides ≥ 2.27 mmol/L, total cholesterol ≥ 6.19 mmol/L, low-density lipoprotein cholesterol ≥ 4.14 mmol/L, and/or high-density lipoprotein cholesterol < 1.04 mmol/L.

Patients with a history of chronic kidney disease or men and women with serum creatinine levels > 105 *μ*mol/L and > 100 *μ*mol/L, respectively, were classified as having chronic kidney disease.

Ischemic stroke was considered based on a history of ischemic stroke, including neurological deficits and/or imaging evidence.

### 2.3. Statistical Analyses

The incidence of CHD was first assessed using a univariate analysis of diabetes and nondiabetes patients in all included populations. Binary logistic regression was then used to establish a multivariate model for the analysis of CHD risk factors. Then, all patients with diabetes were excluded, and blood glucose was grouped according to the ADA, WHO, or other cut-off criteria. Univariate analysis of CHD incidence and baseline data were assessed between the groups. To eliminate the effects of confounding factors, 1 : 1 propensity score matching (PSM) was applied to eliminate the differences between the IFG and normal groups and to calculate for the adjusted odds ratios (OR). The covariates included in the PSM model were age, hypertension, smoking, drinking, dyslipidemia, chronic kidney disease, and previous ischemic stroke. After PSM, each variable was compared to ensure comparability between both groups. Multivariate binary logistic regression was used to explore the relationship between FPG and CHD in different populations. In all univariate analyses of the incidence of CHD, count data were assessed using the chi-squared test. Normally distributed continuous data were expressed are mean ± variance and were compared using the selected independent Student's *t*-test, while nonnormally distributed data are expressed as median and interquartile range and were evaluated using the Mann–Whitney *U* test. Multivariate ordered logistic regression was used to analyze the relationship between IFG and the severity of coronary lesions. Continuous variables were used when analyzing the relationship between CHD and FPG in the different populations. The factors included in all multivariate models were baseline data with statistical differences in the univariate analysis. All statistical analyses, including PSM, were performed with SPSS software 25.0 (IBM, USA). *p* values were 2-tailed, and *p* < 0.05 was considered significant.

## 3. Results

A total of 2,987 patients were enrolled in the study, including 53.8% men (*n* = 1,607), 66.9% with CHD (*n* = 1,997), 22.3% with diabetes (*n* = 665), 20% with IFG-ADA (*n* = 598), and 8.7% with IFG-WHO (*n* = 260).

### 3.1. Effects of Diabetes on CHD

In all populations, univariate analysis showed an increased incidence of CHD in patients with diabetes, with an OR of 2.754 (95% CI = 2.221–3.416, *p* < 0.01). After adjusting for age, sex, hypertension, dyslipidemia, smoking, alcohol consumption, and ischemic stroke in the multivariate model, an adjusted OR of 2.593 (95% CI = 2.067–3.253, *p* < 0.01) was found (Tables 1 and 2).

Given the higher risk ratio of men to women concerning the incidence of CHD, the adjusted OR was 2.153 (95% CI = 1.717–2.700, *p* < 0.01), and the sexes were analyzed separately.

Consistent with the general population, the incidence of CHD was elevated in both diabetic male and female patients. In men, univariate analysis showed that the OR of CHD in patients with diabetes was 3.003 (95% CI = 2.095–4.303, *p* < 0.01). After correcting for age, smoking, hypertension, dyslipidemia, and ischemic stroke history, the adjusted OR was 2.774 (95% CI = 1.913–4.020, *p* < 0.01). In women, the OR was 3.069 (95% CI = 2.327–4.047, *p* < 0.01), and the adjusted OR was 2.496 (95% CI = 1.868–3.335, *p* < 0.01). In the multivariate model, hypertension was a risk factor for CHD in women but not in men. In contrast, smoking was a risk factor for CHD in men but not in women. In fact, the smoking rate of women with CHD was very low (1.11%).

### 3.2. Impact of IFG on CHD

To explore the impact of IFG on morbidity, we excluded patients with diabetes, and the remaining population was divided into groups according to the diagnostic criteria of ADA and WHO, namely, an ADA-defined normal group and IFG group or a WHO-defined normal group and IFG group. They were analyzed separately by sex.

**Figure 1:**
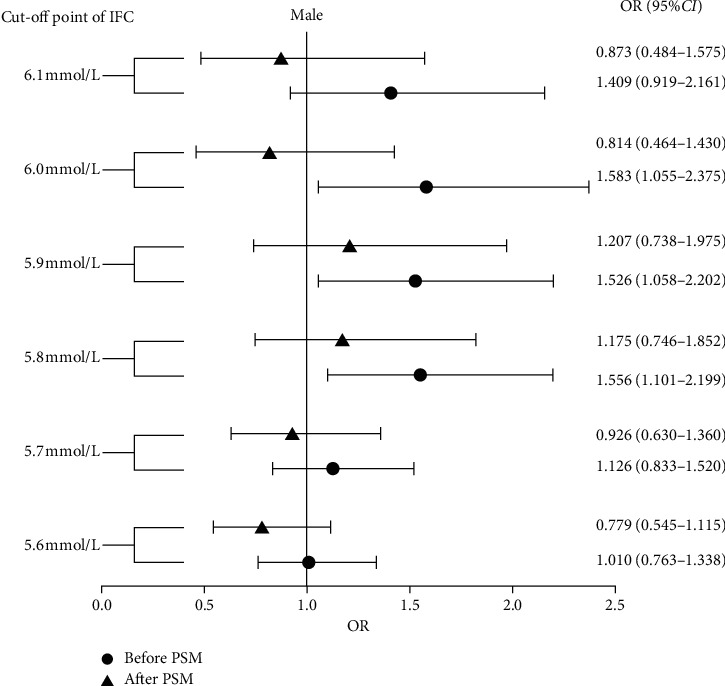
Odds ratio of IFG defined by the different cut-off points in males to the incidence of CHD. CHD: coronary heart disease; CI: confidence interval; IFG: impaired fasting glucose; OR: odds ratio; PSM: propensity score match.

In men, univariate analysis showed an increased incidence of CHD in the WHO-defined IFG group, but the result was not significant (76.6% versus 69.9%, *p* = 0.115). The incidence of CHD in the ADA-defined IFG group was also not significant relative to the normal group (70.5% versus 70.7%, *p* = 0.944). Owing to the decreased sample size in men with FPG greater than 6.1 mmol/L, the degree of difference may have been affected. New cut-off points between 5.6 mmol/L and 6.1 mmol/L at 0.1 mmol/L increments were set to avoid missing potentially important cut-off thresholds. Univariate analysis showed that when the cut-off points were 5.8 mmol/L, 5.9 mmol/L, and 6.0 mmol/L, there were significant differences in the incidence of CHD between the IFG and normal blood glucose groups. After using PSM to rule out confounding factors, the IFG group, without any cut-off points, was justified. Although no differences in baseline data were observed before PSM when using cut-off points 5.6 mmol/L to 5.9 mmol/L, PSM was still used to further reduce the difference in baseline data between the two groups (Table 3 and Figure 1).

**Figure 2:**
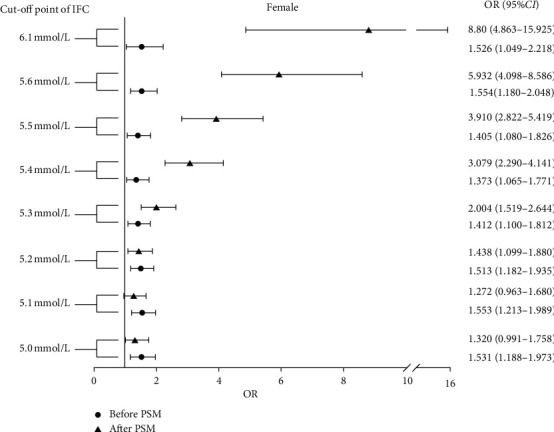
Odds ratio of IFG defined by the different cut-off points in females to the incidence of CHD. CHD: coronary heart disease; CI: confidence interval; IFG: impaired fasting glucose; OR: odds ratio; PSM: propensity score match.

In women, both the ADA- and WHO-defined IFG groups showed significant increases in CHD incidence during univariate analysis. To better eliminate the differences in baseline data, PSM was applied. After adjusting for the confounding factors, significantly increased CHD incidence still remained. To identify whether the difference in the incidence rate already existed at lower FPG levels, a plurality of cut-off points at 5.6 mmol/L or less at intervals of 0.1 mmol/L were set, and PSM was separately performed. There were significant differences in the incidence of CHD between the IFG and normal groups when a 5.2 mmol/L cut-off point was selected (OR = 1.438, 95% CI = 1.099–1.880, *p* = 0.008). At cut-off points 5.0 mmol/L and 5.1 mmol/L, although CHD was higher in the IFG group than in the normal group, it was not significant (Table 4 and Figure 2). The ordered logistic regression analysis revealed that CHD lesions were more severe in the IFG group when the cut-off point was set at 5.2 mmol/L (OR = 1.406, 95% CI = 1.107–1.788, *p* = 0.005, Table 5).

### 3.3. Additional Analysis

To determine the reason why elevated fasting glucose only affected the incidence of CHD in women but not in men, we compared the baseline characteristics of men and women with angiographically confirmed CHD. Relative to female CHD patients, male CHD patients had lower FPG levels, age at onset, and incidence of hypertension but significantly higher dyslipidemia, smoking, and drinking habits. To explore whether the differences between men and women were caused by the different proportions of risk factors, we established 5 multivariate models according to smoking status, drinking habits, dyslipidemia, age (≥60 years old), and hypertension. The effects of FPG on the incidence of CHD occurred in the group without dyslipidemia (adjusted OR = 1.252, 95% CI = 1.042–1.504) and in nonsmokers (adjusted OR = 1.234, 95% CI = 1.038–1.468). This was in line with the higher proportions of hyperlipidemia and smokers in men (Tables 6 and 7).

## 4. Discussion

### 4.1. Fasting Glucose and CHD

In the present investigation, we found that elevated levels of FPG cause an increase in CHD incidence in women from rural areas of northern Henan, China, but not in men. These results are similar to those of the Framingham cohort study [8]. However, a novel finding of our study was that diabetes-free women were associated with a significantly increased incidence of CHD and more severe degrees of CHD when FPG levels were >5.2 mmol/L.

Our results showed several high ORs, especially when the cut-off point was 6.1 mmol/L in men, wherein the OR was 8.80. The possible reason was that, compared with prospective analyses, all patients in this study received coronary angiography, which excludes the effects of false negatives. In prospective cohort studies, there may be patients who actually had CHD but did not visit the hospital for examination, such as in cases of asymptomatic myocardial ischemia. The presence of false negatives may also lead to an underestimation of blood glucose effects on CHD, which was the reason why every patient we selected in this study had to have undergone coronary angiography.

The cut-off point for IFG was redefined in this study, with the cut-off of 5.2 mmol/L lower than that reported by the WHO and ADA. A study in Canada found that even glycemia under the diagnostic criteria of gestational diabetes can increase the risk of cardiovascular disease after several years of exposure [15]. Abnormal glucose metabolism damages blood vessels earlier than originally expected. In addition to the direct damage of vascular endothelial function caused by hyperglycemia, the insulin resistance and hyperinsulinemia already present would promote the formation of atherosclerosis even before the rise in blood glucose levels [16]. However, it may be difficult to maintain fasting glucose levels below 5.2 mmol/L using drugs alone. Excessive drug application may increase the risk of hypoglycemia, which also causes vascular damage [17]. We can, however, improve the body's metabolic state and insulin sensitivity by adjusting lifestyles, increasing exercise, and reducing weight, thereby reducing the risk of CHD [18–20]. It is worth mentioning that the definition of “impaired fasting glucose” in this study only accounted for the incidence and degree of CHD, while the concept of IFG is in fact more complex; for example, whether IFG will develop into clinically diagnosed diabetes in the next few years or whether IFG causes an increase in the incidence of other cardiovascular diseases remains unclear.

### 4.2. Comparison of Risk Factors between Men and Women

Similar to the Framingham cohort study, we found discrepancies in the results between men and women. Elevated FPG levels in men were not associated with an increase in CHD incidence. This may be due to the inherent differences between sexes, such as the differences in chromosomes or in estrogen levels. It may also be the differences in composition of risk factor profiles. In comparison with patients having positive angiographic results in both men and women, male CHD patients had lower FPG levels, age of onset, and rate of hypertension, but higher rates of smoking, drinking, and dyslipidemia; all 3 factors damage the cardiovascular system. Further analysis showed that dyslipidemia and smoking in the population did not affect the incidence of CHD, whereas the opposite was observed in those without dyslipidemia and who were nonsmokers. This was likely due to the higher dyslipidemia and smoking rates in men, which could have masked the damage to blood vessels, caused by elevated FPG levels, and thus may have obscured the effects of IFG on CHD incidence in men. This can partly explain the difference between men and women regarding the impact of IFG on CHD incidence. In rural China, men constitute the primary labor force and are burdened with more physical tasks. Physical activity can improve vascular endothelial function, reduce body inflammatory response and visceral fat, and provide other benefits, as well as limit the incidence of CHD. This may also be the reason why IFG did not affect the incidence of CHD in men [21].

### 4.3. Study Limitations

This project was single-centered, and the selected population was exclusively from the northern region of Henan Province. Thus, the results obtained likely do not represent all rural areas of China. However, there are more than 40 million people living in the rural areas of Henan Province; thus, our research provides critical insight into the health of these individuals.

The Clinical Data and Sample Resource Research Center of Cardiovascular Diseases in the Henan Province has only recently been established, and the available sample size was therefore limited, with existing data consisting mostly of individuals from a rural population. Therefore, it was difficult to compare and analyze the differences between urban and rural populations. However, the database is continuously updated, and more information will be available for future research.

Furthermore, several important data like body mass index and CHD family history were not available in the current database, which impacted the results of our research. The present investigation also did not intend to use FPG as the assessment of diabetes status. There may have been individuals with normal FPG levels but with 2-hour postload glucose levels that fit the diagnostic criteria for diabetes.

## 5. Conclusion

The incidence of CHD increased in diabetes-free women in rural areas of northern Henan when the FPG level was greater than 5.2 mmol/L. It is hence recommended that women in this region be more active in managing their blood glucose levels and should adopt healthy lifestyle interventions as early as possible, especially individuals with other risk factors. Although the incidence of CHD in men was not affected by the increase in FPG, risk factors, such as smoking, should be limited to control blood lipid levels.

## Figures and Tables

**Table 1:** Baseline characteristics of patients with and without CHD.

	All participants		*p* value	Male		*p* value	Female		*p* value
	CHD (*n* = 1997)	No CHD (*n* = 990)		CHD (*n* = 1187)	No CHD (*n* = 420)		CHD (*n* = 810)	No CHD (*n* = 570)	
Diabetes	546 (27.3%)	119 (12.0%)	<0.001	273 (20.0%)	38 (9.0%)	<0.001	273 (29.3%)	81 (14.2%)	<0.001
Age	61 (53–67)	54 (48–62)	<0.001	59 (51–66)	52 (46–60)	<0.001	62 ± 9	57 ± 9	<0.001
Male	1187 (59.4%)	420 (42.4%)	<0.001	/	/	/	/	/	/
Smoking	793 (39.7%)	257 (26.0%)	<0.001	784 (66.0%)	252 (60.0%)	0.026	9 (1.1%)	5 (0.9%)	0.669
Drinking	404 (20.2%)	147 (14.8%)	<0.001	398 (33.5%)	143 (34.0%)	0.847	6 (0.7%)	4 (0.7%)	0.933
Hypertension	1442 (72.2%)	623 (62.9%)	<0.001	796 (67.1%)	254 (60.5%)	0.015	646 (79.8%)	369 (64.7%)	<0.001
Dyslipidemia	1029 (51.5%)	373 (37.7%)	<0.001	668 (56.3%)	187 (44.5%)	<0.001	361 (44.6%)	186 (32.6%)	<0.001
Previous ischemic stroke	263 (13.2%)	70 (7.1%)	<0.001	160 (13.5%)	23 (5.5%)	<0.001	103 (12.7%)	47 (8.2%)	0.009
Chronic kidney disease	20 (1.0%)	8 (0.8%)	0.606	15 (1.3%)	4 (1.0%)	0.612	5 (0.6%)	4 (0.7%)	0.848

CHD: coronary heart disease.

**Table 2:** Multivariate analysis of risk factors for coronary heart disease.

	All participants		*p* value	Male		*p* value	Female		*p* value
	B	OR (95% CI)		B	OR (95% CI)		B	OR (95% CI)	
Diabetes	0.953	2.593(2.067–3.253)	<0.001	1.02	2.774(1.913–4.020)	<0.001	0.915	2.496(1.868–3.335)	<0.001
Age	0.053	1.054 (1.045–1.064)	<0.001	0.049	1.051 (1.038–1.063)	<0.001	0.058	1.059 (1.046–1.073)	<0.001
Male	0.767	2.153 (1.717–2.700)	<0.001	/	/	/	/	/	/
Smoking	0.307	1.360 (1.058–1.748)	0.016	0.286	1.332 (1.046–1.695)	0.020	/	/	/
Drinking	−0.032	0.968 (0.748–1.254)	0.808	/	/	/	/	/	/
Hypertension	0.313	1.367 (1.145–1.632)	0.001	0.097	1.102 (0.863–1.407)	0.435	0.547	1.727 (1.333–2.239)	<0.001
Dyslipidemia	0.482	1.620 (1.369–1.917)	<0.001	0.539	1.715 (1.351–2.176)	<0.001	0.411	1.508 (1.187–1.915)	0.001
Previous ischemic stroke	0.273	1.315 (0.980–1.764)	0.068	0.604	1.829 (1.144–2.926)	0.012	0.024	1.024 (0.694–1.511)	0.906

CI: confidence interval; OR: odds ratio.

**Table 3:** Baseline characteristics of IFG and normal groups defined by different cut-off points before and after PSM in male patients.

Cut-off point of IFG			Age	Hypertension	Smoking	Drinking	Dyslipidemia	Chronic kidney disease	Previous ischemic stroke	CHD
5.6 mmol/L	Before PSM	NG (*n* = 989)	57 (49–64)	622 (62.9%)	646 (65.3%)	341 (34.5%)	487 (49.2%)	11 (1.1%)	108 (10.9%)	697 (70.5%)
		IFG (*n* = 307)	57 (50–64)	200 (65.1%)	200 (65.1%)	96 (31.3%)	163 (53.1%)	7 (2.3%)	24 (7.8%)	217 (70.7%)
		*p* value	0.469	0.474	0.956	0.299	0.238	0.159	0.116	0.944
	After PSM	NG (*n* = 306)	57 ± 11	188 (61.4%)	198 (64.7%)	80 (26.1%)	151 (49.3%)	4 (1.3%)	38 (12.4%)	231 (75.5%)
		IFG (*n* = 306)	57 ± 10	199 (65%)	200 (65.4%)	96 (31.4%)	162 (52.9%)	7 (2.3%)	24 (7.8%)	216 (70.6%)
		*p* value	0.703	0.356	0.865	0.153	0.374	0.361	0.061	0.172
5.7 mmol/L	Before PSM	NG (*n* = 1031)	57 (49–64)	648 (62.9%)	671 (65.1%)	350 (33.9%)	512 (49.7%)	12 (1.2%)	111 (10.8%)	722 (70%)
		IFG (*n* = 265)	56 (50–64)	174 (65.7%)	175 (66%)	87 (32.8%)	138 (52.1%)	6 (2.3%)	21 (7.9%)	192 (72.5%)
		*p* value	0.748	0.397	0.771	0.731	0.483	0.233	0.173	0.440
	After PSM	NG (*n* = 265)	58 ± 11	158 (59.6%)	176 (66.4%)	80 (30.2%)	116 (43.8%)	2 (0.8%)	33 (12.5%)	196 (74%)
		IFG (*n* = 265)	57 ± 10	174 (65.7%)	175 (66%)	87 (32.8%)	138 (52.1%)	6 (2.3%)	21 (7.9%)	192 (72.5%)
		*p* value	0.493	0.151	0.927	0.513	0.056	0.285	0.085	0.695
5.8 mmol/L	Before PSM	NG (*n* = 1081)	57 (49–64)	679 (62.8%)	700 (64.8%)	365 (33.8%)	538 (49.8%)	13 (1.2%)	112 (10.4%)	747 (69.1%)
		IFG (*n* = 215)	58 (51–65)	143 (66.5%)	146 (67.9%)	72 (33.5%)	112 (52.1%)	5 (2.3%)	20 (9.3%)	167 (77.7%)
		*p* value	0.096	0.304	0.375	0.938	0.534	0.202	0.639	0.012
	After PSM	NG (*n* = 204)	57 ± 9	136 (66.7%)	142 (69.6%)	65 (31.9%)	108 (52.9%)	1 (0.5%)	20 (9.8%)	152 (74.5%)
		IFG (*n* = 204)	57 ± 10	136 (66.7%)	141 (69.1%)	70 (34.3%)	107 (52.5%)	5 (2.5%)	19 (9.3%)	158 (77.5%)
		*p* value	0.987	1	0.914	0.599	0.921	0.215	0.866	0.487
5.9 mmol/L	Before PSM	NG (*n* = 1109)	57 (49–64)	693 (62.5%)	713 (64.3%)	371 (33.5%)	555 (50%)	14 (1.3%)	113 (10.2%)	769 (69.3%)
		IFG (*n* = 187)	58 (51–65)	129 (69%)	133 (71.1%)	66 (35.3%)	95 (50.8%)	4 (2.1%)	19 (10.2%)	145 (77.5%)
		*p* value	0.069	0.088	0.070	0.622	0.848	0.314	0.990	0.023
	After PSM	Normal (*n* = 166)	57 ± 9	116 (69.9%)	120 (72.3%)	53 (31.9%)	90 (54.2%)	0 (0%)	11 (6.6%)	120 (72.3%)
		IFG (*n* = 166)	57 ± 9	116 (69.9%)	120 (72.3%)	60 (36.1%)	90 (54.2%)	3 (1.8%)	11 (6.6%)	126 (75.9%)
		*p* value	1	1	0.720	0.417	1	0.248	1	0.452
6 mmol/L	Before PSM	NG (*n* = 1144)	57 (49–64)	716 (62.6%)	738 (64.5%)	380 (33.2%)	571 (49.9%)	15 (1.3%)	118 (10.3%)	795 (69.5%)
		IFG (*n* = 152)	60 (51–66)	106 (69.7%)	108 (71.1%)	57 (37.5%)	79 (52%)	3 (2.0%)	14 (9.2%)	119 (78.3%)
		*p* value	0.013	0.086	0.111	0.294	0.633	0.459	0.672	0.025
	After PSM	NG (*n* = 152)	60 ± 9	98 (64.5%)	106 (69.7%)	45 (29.6%)	65 (42.8%)	0 (0%)	23 (15.1%)	124 (81.6%)
		IFG (*n* = 152)	59 ± 10	106 (69.7%)	108 (71.1%)	57 (37.5%)	79 (52%)	3 (2%)	14 (9.2%)	119 (78.3%)
		*p* value	0.100	0.329	0.802	0.145	0.108	0.248	0.114	0.474
6.1 mmol/L	Before PSM	NG (*n* = 1168)	57 (49–64)	730 (62.5%)	754 (64.6%)	388 (33.2%)	583 (49.9%)	16 (1.4%)	119 (10.2%)	816 (69.9%)
		IFG (*n* = 128)	61 (51–66)	92 (71.9%)	92 (71.9%)	49 (38.3%)	67 (52.3%)	2 (1.6%)	13 (10.2%)	98 (76.6%)
		*p* value	0.023	0.037	0.099	0.250	0.602	0.696	0.991	0.115
	After PSM	NG (*n* = 128)	60 (51–67)	92 (71.9%)	83 (64.8%)	37 (28.9%)	57 (44.5%)	1 (0.8%)	23 (18%)	101 (78.9%)
		IFG (*n* = 128)	61 (51–66)	92 (71.9%)	92 (71.9%)	49 (38.3%)	67 (52.3%)	2 (1.6%)	13 (10.2%)	98 (76.6%)
		*p* value	0.708	1	0.226	0.112	0.211	1	0.072	0.652

CHD: coronary heart disease; IFG: impaired fasting glucose; NG: normal glucose; PSM: propensity score match.

**Table 4:** Baseline characteristics of IFG and normal groups defined by different cut-off points before and after PSM in female patients.

Cut-off point of IFG			Age	Hypertension	Smoking	Drinking	Dyslipidemia	Chronic kidney disease	Previous ischemic stroke	CHD
5 mmol/L	Before PSM	NG (*n* = 387)	56 (50–65)	262 (67.7%)	3 (0.8%)	3 (0.8%)	128 (33.1%)	3 (0.8%)	37 (9.6%)	177 (45.7%)
		IFG (*n* = 639)	61 (53–66)	461 (72.1%)	9 (1.4%)	6 (0.9%)	236 (36.9%)	4 (0.6%)	49 (7.7%)	360 (56.3%)
		*p* value	<0.001	0.130	0.551	1	0.211	1	0.289	0.001
	After PSM	NG (*n* = 376)	57 (51–65)	252 (67%)	3 (0.8%)	3 (0.8%)	126 (33.5%)	3 (0.8%)	34 (9%)	167 (44.4%)
		IFG (*n* = 376)	57 (52–65)	259 (68.9%)	2 (0.5%)	2 (0.5%)	146 (38.8%)	1 (0.3%)	34 (9%)	193 (51.3%)
		*p* value	0.755	0.584	1	1	0.129	0.624	1	0.058
5.1 mmol/L	Before PSM	NG (*n* = 462)	57 (50∼65)	309 (66.9%)	3 (0.6%)	3 (0.6%)	152 (32.9%)	3 (0.6%)	41 (8.9%)	214 (46.3%)
		IFG (*n* = 564)	61 (53–67)	414 (73.4%)	9 (1.6%)	6 (1.1%)	212 (37.6%)	4 (0.7%)	45 (8.0%)	323 (57.3%)
		*p* value	<0.001	0.023	0.161	0.525	0.118	1	0.606	< 0.001
	After PSM	NG (*n* = 400)	60 (52–65)	289 (72.3%)	1 (0.3%)	2 (0.5%)	133 (33.3%)	3 (0.8%)	41 (10.3%)	179 (44.8%)
		IFG (*n* = 400)	60 (53–65)	264 (66.0%)	5 (1.3%)	5 (1.3%)	141 (35.3%)	3 (0.8%)	29 (7.3%)	203 (50.8%)
		*p* value	0.523	0.056	0.217	0.255	0.551	1	0.133	0.089
5.2 mmol/L	Before PSM	NG (*n* = 528)	58 (51–65)	357 (67.6%)	6 (1.1%)	4 (0.8%)	181 (34.3%)	3 (0.6%)	42 (8.0%)	250 (47.3%)
		IFG (*n* = 498)	61 (53–67)	366 (73.5%)	6 (1.2%)	5 (1.0%)	183 (36.7%)	4 (0.8%)	44 (8.8%)	287 (57.6%)
		*p* value	<0.001	0.039	0.919	0.747	0.409	0.718	0.611	0.001
	After PSM	NG (*n* = 431)	60 (53–66)	318 (73.8%)	4 (0.9%)	2 (0.5%)	145 (33.6%)	3 (0.7%)	40 (9.3%)	194 (45.0%)
		IFG (*n* = 431)	60 (53–65)	305 (70.8%)	5 (1.2%)	5 (1.2%)	153 (35.5%)	4 (0.9%)	37 (8.6%)	233 (54.1%)
		*p* value	0.709	0.323	1	0.451	0.567	1	0.720	0.008
5.3 mmol/L	Before PSM	NG (*n* = 595)	58 (51–65)	406 (68.2%)	7 (1.2%)	5 (0.8%)	204 (34.3%)	3 (0.5%)	49 (8.2%)	290 (48.7%)
		IFG (*n* = 431)	61 (53–67)	317 (73.5%)	5 (1.2%)	4 (0.9%)	160 (37.1%)	4 (0.9%)	37 (8.6%)	247 (57.3%)
		*p* value	<0.001	0.065	0.981	1	0.348	0.462	0.842	0.007
	After PSM	NG (*n* = 414)	60 ± 9	285 (68.8%)	3 (0.7%)	2 (0.5%)	138 (33.3%)	3 (0.7%)	38 (9.2%)	160 (38.6%)
		IFG (*n* = 414)	60 ± 9	303 (73.2%)	5 (1.2%)	4 (1.0%)	153 (37.0%)	4 (1.0%)	35 (8.5%)	231 (55.8%)
		*p* value	0.981	0.168	0.725	0.686	0.275	1	0.713	< 0.001
5.4 mmol/L	Before PSM	NG (*n* = 640)	58 (52–65)	439 (68.6%)	8 (1.3%)	5 (0.8%)	215 (33.6%)	3 (0.5%)	53 (8.3%)	316 (49.4%)
		IFG (*n* = 386)	61 (53–67)	284 (73.6%)	4 (1.0%)	4 (1.0%)	149 (38.6%)	4 (1.0%)	33 (8.5%)	221 (57.3%)
		*p* value	<0.001	0.090	1	0.735	0.104	0.436	0.881	0.014
	After PSM	NG (*n* = 386)	60 (53–67)	282 (73.1%)	4 (1.0%)	2 (0.5%)	144 (37.3%)	3 (0.8%)	32 (8.3%)	117 (30.3%)
		IFG (*n* = 386)	61 (53–67)	284 (73.6%)	4 (1.0%)	4 (1.0%)	149 (38.6%)	4 (1.0%)	33 (8.5%)	221 (57.3%)
		*p* value	0.204	0.871	1	0.686	0.711	1	0.897	< 0.001
5.5 mmol/L	Before PSM	NG (*n* = 688)	59 (52–65)	467 (67.9%)	8 (1.2%)	5 (0.7%)	236 (34.3%)	3 (0.4%)	53 (7.7%)	341 (49.6%)
		IFG (*n* = 338)	61 (54–68)	256 (75.7%)	4 (1.2%)	4 (1.2%)	128 (37.9%)	4 (1.2%)	33 (9.8%)	196 (58.0%)
		*p* value	<0.001	0.009	1	0.487	0.262	0.227	0.263	0.011
	After PSM	NG (*n* = 336)	61 (53–67)	264 (78.6%)	4 (1.2%)	2 (0.6%)	105 (31.3%)	3 (0.9%)	33 (9.8%)	87 (25.9%)
		IFG (*n* = 336)	61 (54–68)	254 (75.6%)	4 (1.2%)	4 (1.2%)	127 (37.8%)	4 (1.2%)	32 (9.5%)	194 (57.7%)
		*p* value	0.660	0.359	1	0.686	0.074	1	0.896	< 0.001
5.6 mmol/L	Before PSM	NG (*n* = 735)	59 (52–65)	499 (67.9%)	8 (1.1%)	6 (0.8%)	255 (34.7%)	3 (0.4%)	56 (7.6%)	362 (49.3%)
		IFG (*n* = 291)	61 (54–68)	224 (77.0%)	4 (1.4%)	3 (1.0%)	109 (37.5%)	4 (1.4%)	30 (10.3%)	175 (60.1%)
		*p* value	< 0.001	0.004	0.749	0.719	0.404	0.105	0.161	0.002
	After PSM	NG (*n* = 291)	61 (53–67)	234 (80.4%)	4 (1.4%)	2 (0.7%)	100 (34.4%)	2 (0.7%)	27 (9.3%)	59 (20.3%)
		IFG (*n* = 291)	61 (54–68)	224 (77.0%)	4 (1.4%)	3 (1.0%)	109 (37.5%)	4 (1.4%)	30 (10.3%)	175 (60.1%)
		*p* value	0.531	0.311	1	1	0.437	0.686	0.781	< 0.001
6.1 mmol/L	Before PSM	NG (*n* = 894)	59 (52–65)	620 (69.4%)	9 (1.0%)	7 (0.8%)	313 (35.0%)	7 (0.8%)	71 (7.9%)	456 (51.0%)
		IFG (*n* = 132)	62 (55–68)	103 (78.0%)	3 (2.3%)	2 (1.5%)	51 (38.6%)	0 (0.0%)	15 (11.4%)	81 (61.4%)
		*p* value	0.002	0.041	0.193	0.326	0.416	0.604	0.185	0.026
	After PSM	NG (*n* = 130)	61 ± 9	103 (79.2%)	2 (1.5%)	0 (0%)	51 (39.2%)	1 (0.8%)	9 (6.9%)	20 (15.4%)
		IFG (*n* = 130)	61 ± 9	101 (77.7%)	3 (2.3%)	2 (1.5%)	51 (39.2%)	0 (0.0%)	14 (10.8%)	80 (61.5%)
		*p* value	0.861	0.763	1	0.498	1	1	0.275	< 0.001

CHD: coronary heart disease; IFG: impaired fasting glucose; NG: normal glucose; PSM: propensity score match.

**Table 5:** Relationship between IFG defined by 5.2 mmol/L and degree of lesion.

	Severity of coronary lesions #				
	Normal	Mild lesions	Moderate lesions	Severe lesions	Total
IFG (5.2 mmol/L)	56 (37.09%)	261 (46.94%)	115 (54.76%)	66 (60.55%)	498
Total	151	556	210	109	1026
OR (95% CI)	1.660 (1.311–2.102), *p* < 0.001				
Adjust OR (95% CI)^*∗*^	1.406 (1.107–1.788), *p* = 0.005				

^*∗*^Adjusted for age, hypertension, dyslipidemia, and previous ischemic stroke. #Parallel line test shows *p* = 0.120. CI: confidence interval; IFG: impaired fasting glucose; OR: odds ratio.

**Table 6:** Differences in baseline characteristics between sexes in patients with coronary heart disease.

	Age	Hypertension	Dyslipidemia	Smoking	Drinking	Previous ischemic stroke	Chronic kidney disease	FPG
Male (*n* = 914)	59 (51–66)	599 (65.5%)	479 (52.4%)	612 (67.0%)	306 (33.5%)	113 (12.4%)	15 (1.6%)	5.07 (4.68–5.56)
Female (*n* = 537)	62 (54–68)	422 (78.6%)	215 (40.0%)	9 (1.7%)	6 (1.1%)	55 (10.2%)	3 (0.6%)	5.25 (4.86–5.74)
*p*	<0.001	<0.001	<0.001	<0.001	<0.001	0.223	0.072	<0.001

FPG: fasting plasma glucose.

**Table 7:** Relationship between continuous variable fasting blood glucose data and incidence of coronary heart disease in different population classifications.

	Dyslipidemia		Hypertension		Smoking		Drinking		Old	
	0	+	0	+	0	+	0	+	0	+
Adjust OR (95% CI)^*∗*^	1.252 (1.042–1.504)	0.956 (0.775–1.179)	1.269 (0.986–1.634)	1.059 (0.898–1.250)	1.234 (1.038–1.468)	0.941 (0.750–1.179)	1.003 (0.735–1.370)	1.140 (0.980–1.327)	1.129 (0.948–1.345)	1.192 (0.956–1.486)
*p*	0.017	0.675	0.065	0.497	0.017	0.595	0.983	0.090	0.172	0.119

^*∗*^Multivariate binary logistic regression adjusted for differences in univariate analysis between groups. “0” for negative and “+” for positive. CI: confidence interval; OR: odds ratio.

## Data Availability

The data used to support the findings of this study are available from the corresponding author upon request.
